# The association between tobacco or nicotine product use behaviors and non-compliance with mask-wearing during the COVID-19 pandemic: a cross-sectional study in Korea

**DOI:** 10.4178/epih.e2022087

**Published:** 2022-10-07

**Authors:** Da-eun Lee, Heewon Kang, Sung-il Cho

**Affiliations:** 1 Department of Public Health Science, Graduate School of Public Health, Seoul National University, Seoul, Korea; 2 Medical Intensive Care Unit, Seoul ST. Mary’s Hospital, The Catholic University of Korea, Seoul, Korea; 3 Institute of Health and Environment, Seoul National University, Seoul, Korea

**Keywords:** Health behavior, Masks, COVID-19, Respiratory tract infections, Communicable disease control, Smoking prevention

## Abstract

**OBJECTIVES:**

It is necessary to investigate tobacco or nicotine product (TNP) use which acts as a risk factor for coronavirus disease 2019 (COVID-19) infection. Especially, wearing a mask is difficult to practice while using TNP. Therefore, this study aimed to examine the association between TNP use behaviors and non-compliance with mask-wearing during the COVID-19 pandemic.

**METHODS:**

The samples of 208,618 Korean adults from 2020 Community Health Survey in Korea were used. As an independent variable, TNP use behaviors such as TNP use status, changes in TNP use after the COVID-19 outbreak, TNP types, and attempt to quit were analyzed. Logistic regression was performed on gender-stratified participants.

**RESULTS:**

Among men, the odds ratio (OR) of current and former TNP users were 2.00 (95% confidence interval [CI], 1.66 to 2.40) and 1.32 (95% CI, 1.09 to 1.60), respectively, compared to never users. In women, OR was 1.50 (95% CI, 1.00 to 2.26) for former users. Cigarette use was more associated with not wearing a mask than non-cigarette tobacco or nicotine product (NCTNP) use (OR, 1.53; 95% CI, 1.12 to 2.08). Men whose TNP use decreased had lower non-compliance (OR, 0.52; 95% CI, 0.36 to 0.74); while women whose TNP use increased had lower non-compliance (OR, 0.13; 95% CI, 0.07 to 0.26).

**CONCLUSIONS:**

Current and former users were less likely to wear masks. Cigarette use was more associated with not wearing a mask than NCTNP use. Changes in TNP use showed association for men and women; however, in the opposite direction. Therefore, more attention should be paid to TNP use prevention and cessation support during the epidemic of respiratory infectious diseases. Moreover, it is necessary to identify risk factors of cigarette users in compliance with mask-wearing.

## GRAPHICAL ABSTRACT

[Fig f2-epih-44-e2022087]

## INTRODUCTION

Smoking is a significant risk factor for various cancers, coronary artery diseases, cardiovascular diseases, and atherosclerotic peripheral vascular disease. It is the most critical risk factor for chronic obstructive pulmonary disease [1]. The number of deaths worldwide from smoking is more than 8 million annually, and the economic burden is considerable [2].

According to the Organization for Economic Cooperation and Development (OECD), the average proportion of daily smokers (age ≥ 15 years) among OCED countries was 16.5% in 2019; Korea was ranked 21st among 38 countries at 16.4% [3]. The proportion of daily smokers among men in Korea is 28.5%, far higher than the OECD average (20.6%), which ranks ninth among 43 countries [4]. It is noteworthy that a greater percentage of men (32.6%) smoke than women (6.5%) worldwide [5], as well as in Korea.

As a global response to the seriousness of smoking, the World Health Organization (WHO) established and promoted the Framework Convention on Tobacco Control (FCTC) on February 27, 2005. More than 90% of the world’s population is subject to tobacco control policies [6], and Korea has followed international agreements since it was ratified in May 2005. Korea has also enacted the National Health Promotion Act, established policies based on legal grounds, and conducted various projects and research.

The importance of smoking cessation is rising due to the coronavirus disease 2019 (COVID-19) outbreak on December 2019. As COVID-19 spreads through droplets and causes severe pneumonia, smokers with poor hygiene habits and lung function have a higher risk of contracting COVID-19 with aggressive progression [7,8]. Moreover, contaminated hands and cigarettes come into contact with the mouth while smoking, providing an opportunity to spread COVID-19 [7]. Smoking produces smoke to be exhaled and droplets containing the severe acute respiratory syndrome coronavirus 2 through coughing and sneezing, thus acting as a transmission route for COVID-19 not only to nearby smokers but also to second-hand smokers [7]. Therefore, for public health, the importance of smoking cessation should be addressed during the COVID-19 pandemic [9].

The importance of social distancing and wearing a mask to block the spread of COVID-19 has already been revealed through several studies. Therefore, the higher the rate of mask-wearing, the lower the rate of COVID-19 infection [10]. Social distancing also lowered the number of COVID-19 infections and slowed the spread of COVID-19 [11]. In Korea, wearing a mask indoors and outdoors is mandatory if a distance of 2 m or more cannot be maintained. As an exception, wearing a mask is difficult while smoking, so it is stipulated to keep distance and to wear it before and after smoking [12]. However, even in Hong Kong [13], where the rate of mask-wearing is high, 32.4% of smokers did not wear a mask immediately after smoking, and 74.3% of smokers did not follow social distancing while smoking [14]. In other words, the risk of virus transmission to nearby smokers and non-smokers during and after smoking can be speculated. It is necessary to figure out the non-compliance with mask-wearing by tobacco or nicotine products (TNP) users during the COVID-19 epidemic in Korea, where the smoking rate is high. Therefore, we aimed to analyze the relationship between TNP use behaviors and non-compliance with mask-wearing during the COVID-19 pandemic to further raise the importance of preventing TNP use and support for smoking cessation activities, in a situation where respiratory infectious disease outbreaks are repetitive and to improve quarantine regulation.

## MATERIALS AND METHODS

### Study population

This is a secondary data analysis using raw data from the Korea Disease Control and Prevention Agency’s 2020 Community Health Survey in Korea. The data were collected by trained surveyors using a 1:1 electronic survey from adults aged ≥ 19, living in the sample household from August 16 to October 31, 2020. Out of the 229,269 survey results, data from 208,618 participants were used for the analysis; excluding data with missing values such as ‘don’t know,’ ‘responses rejected,’ and ‘responses not answered’ to the questions considered as variables in this study.

### Measures

#### Tobacco/nicotine product use behaviors

TNP use behaviors included TNP use status, changes in TNP use after the COVID-19 outbreak, TNP types, and attempt to quit. The current use of TNPs was determined based on ‘current’ or ‘for the last one month.’ Those who do not use TNPs currently but used it in the past were classified as former users, and those with no experience of use in a lifetime were classified as never users. Changes in TNP use presented the increase or decrease of use compared to before the COVID-19 pandemic; cases that had not used TNPs since before the outbreak of COVID-19 were treated as ‘not applicable.’ TNP included cigarettes, electronic cigarettes (e-cigarettes, Electronic Nicotine Delivery Systems), and heating tobacco products (HTPs) and excluded other types of cigarettes such as Snus, water pipe tobacco (Shisha or Hookah), and cigars, because these types of cigarettes were not surveyed in 2020. To determine whether or not the participant tried to quit TNP use, the current user was asked if they had quit TNP use for more than one day in the past year.

#### Tobacco/nicotine product use status

With the rapid change in the tobacco or nicotine product market, TNPs with various types, flavors, designs, and methods of use have been released. Accordingly, methods for designating and classifying TNPs have also been used in multiple ways. Classification methods and definitions differ depending on studies, such as conventional tobacco products versus non-conventional tobacco products or combustible cigarettes versus smokeless tobacco products.

In this study, to examine the overall frequency of each cigarette or nicotine product used, users were classified into the following seven groups and compared: cigarette single user, HTP single user, electronic cigarette (e-cigarette) single user, cigarette and HTP dual user, HTP and e-cigarette dual users, cigarette and e-cigarette dual users, and triple users. In the first regression analysis, all participants were classified into current, former, and never users to be compared according to TNP use. In the regression analysis on current users, they were classified into ‘cigarette users’ and ‘noncigarette tobacco or nicotine product (NCTNP) users’ to be compared as to non-compliance with mask-wearing owing to differences in behavior. NCTNP users included HTP or e-cigarette users, and cigarette users who used HTPs or e-cigarettes in combination.

#### Mask-wearing

Wearing a mask was separately investigated indoors and outdoors. In the case of indoors, the question was, ‘Did you wear a mask in an indoor facility used by the unspecified public (example omitted)?’ In the case of outdoors, the question was, ‘Did you wear a mask outdoors when it was difficult to keep a distance of more than 2 m between people?’ The answers were classified as ‘mask-wearing’ when answered as ‘strongly agree’ and ‘agree,’ and ‘not wearing a mask’ when answered as ‘disagree’, respectively. In this study, participants who wore a mask both indoors and outdoors were classified as ‘mask-wearing’, and those who did not wear a mask indoors or outdoors were classified as ‘not wearing a mask’.

#### Other covariates

All analyses were stratified by gender and the adjusted sociodemographic characteristics variables were age, occupation, number of household members, education level, region of residence, marital status, and monthly household income. These variables were confirmed from previous studies about having relationships with exposure and explanatory variables respectively.

In 2020, the proportion of daily smokers was 27.8% for men and 3.9% for women among people aged ≥ 15 years in Korea, indicating a significant gender difference [4]. The main cause of the gap in smoking between men and women was the distinction in smoking behavior [15]. Occupation, education level, and income also were associated with smoking behavior [16]; divorced and single people were less likely to quit smoking [17].

Women wore masks more than men, and the odds ratio (OR) of wearing masks versus not wearing a mask decreased with a decrease in age [18]. Although the rate of mask-wearing was similar in cities and suburbs, it was significantly lower in rural areas [18]. Having a job and working outside were more related to non-compliance with mask-wearing than not having a job and working from home [19]. In the same study, lower education levels were also associated with non-compliance with mask-wearing; people with high income and who were married were more likely to wear masks [20].

### Statistical analysis

Samples were extracted under a complex sampling design, so weights, clusters, and strata were considered to represent the population when estimating the mean and variance. For statistical analysis, the significance level was set at p-value < 0.05, and SAS version 9.4 (survey logistic procedure; SAS Institute Inc., Cary, NC, USA) was used. Logistic regression analysis was conducted twice. First, the non-compliance with mask-wearing of all participants was analyzed by the status of TNP use. Second, the non-compliance with mask-wearing of current users was analyzed by the type of TNP, changes in TNP use after the COVID-19 outbreak, and attempt to quit. Moreover, the analysis was stratified into men and women because of the difference in the smoking rate. The age was used as a continuous variable after confirming the linear shape. OR derived from each regression analysis means the odds of not wearing a mask to the odds of wearing a mask.

In addition, multicollinearity between independent variables was excluded by calculating variance inflation factors. Akaike information criterion (AIC) was compared to test the model fit, and the model with the lowest AIC was used for logistic regression analyses.

### Ethics statement

This study was exempted from the review on May 6, 2022, by the Institutional Review Board of Seoul National University (IRB No. E2205/002-005).

## RESULTS

Table 1 presents the rate of not wearing a mask by socio-demographic characteristics of all participants. Of all men and women, 1.27% and 0.74%, respectively did not wear a mask, indicating a high rate of the practice of wearing a mask. However, the rate of men for non-compliance with mask-wearing was 1.72 times higher than one of women, showing a gender difference. The rate of not wearing a mask among men generally increased with age. The rate of not wearing a mask for women over 70 years old was 1.57%, which was higher than that of other age groups. In the comparison by occupations, the rate of non-compliance with mask-wearing was highest in skilled agricultural and fishery workers for both men and women. Single-person households had a higher rate of not wearing a mask than non-single-person households. Generally, the lower the education level, the higher the rate of non-compliance with mask-wearing, and people living in rural were more likely to avoid wearing a mask than people living in urban. Married men and unmarried women had a higher rate of not wearing a mask, showing contradictory results. For both men and women, the lower the income level, the higher the rate of non-compliance with mask-wearing.

Table 2 present the rate of non-compliance with mask-wearing by TNP use behaviors according to gender. Regarding TNP use status, most men were former users (36.18%), and most women were never users (94.55%). The rate of not wearing a mask was highest among triple users for both men and women. For both men and women, the number of people who decreased TNP use after the COVID-19 outbreak was slightly more than those who increased. Men with increased TNP use had a higher rate of non-compliance with mask-wearing than men with decreased TNP use. Contrarily, among both men and women, the number of participants who did not try to quit TNP use was slightly more than those who tried to quit TNP use. Men who did not try to quit TNP use had a higher rate of not wearing a mask than those who did.

**Figure 1.**
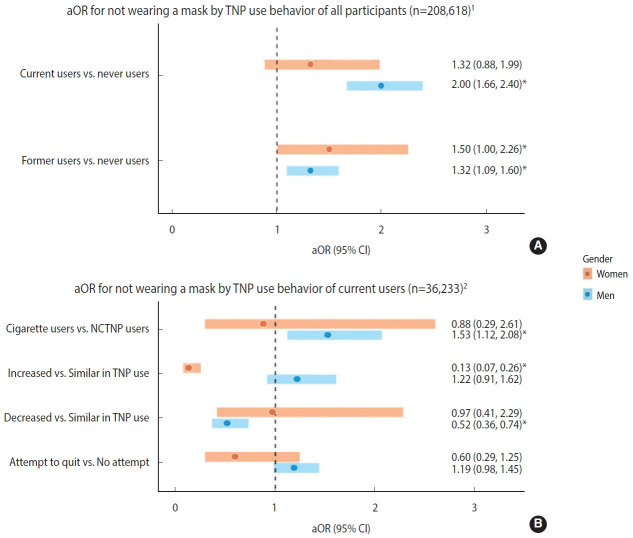
Figure caption

The result of the logistic regression analysis for non-compliance with mask-wearing can be found in Figure 1. When examining the adjusted ORs for not wearing a mask by TNP use behavior of all participants (Figure 1A), the significance was mainly found in men. Among men, the odds of not wearing a mask for current users were 2.00 times higher than for never users (OR, 2.00; 95% confidence interval [CI], 1.66 to 2.40). The odds for former users were 1.32 times higher than those for never users (OR, 1.32; 95% CI, 1.09 to 1.60). Among women, the odds of not wearing a mask were 1.50 times higher for former users than never users (OR, 1.50; 95% CI, 1.00 to 2.26). Figure 1B presents the adjusted ORs for not wearing a mask by TNP use behavior of current users. Among men, cigarette users compared to NCTNP users were associated with not wearing a mask (OR, 1.53; 95% CI, 1.12 to 2.08). A significant difference between the two groups of women was absent. Changes in TNP use after the COVID-19 outbreak were different between men and women. In men, a negative association between the decreased use of TNP and non-compliance with mask-wearing (OR, 0.52; 95% CI, 0.36 to 0.74) was observed. In contrast, in women, a negative association between increased use of TNP and non-compliance with mask-wearing (OR, 0.13; 95% CI, 0.07 to 0.26) was observed. Significant differences in attempt to quit in both men and women were not present. The results of non-compliance with mask-wearing according to the socio-demographic variables are attached as an appendix (Supplementary Materials 1 and 2).

## DISCUSSION

### Main findings

This study investigated TNP use behaviors and non-compliance with mask-wearing during the COVID-19 pandemic in Korea.

Compared to the pre-COVID-19 pandemic, the number of people who decreased TNP use was slightly more than those who increased. This result is contrary to studies conducted in the Netherlands or the United Kingdom [21,22] that the increase in smoking was greater than the decrease, but similar to the study result conducted in Italy that the decrease in smoking was greater than the increase [23]. COVID-19 seems to affect both the increase and decrease of TNP use.

The rate of mask-wearing was 98.73% for men (1.27% of non-compliance with mask-wearing) and 99.26% for women (0.74% of non-compliance with mask-wearing) in Korea. The rate is very high compared to the one in Northern Europe (15% or less) [15] and even compared to Spain (96.4%) and Italy (93.9%), which are known to be high in the rate of mask-wearing among European countries [24]. Among 28 countries, Korea ranked first in the rate of mask-wearing [25], and several pieces of literature explain the reasons as collectivism and previous experience of wearing a mask [26].

However, a difference in non-compliance with mask-wearing by TNP use behavior existed even in such a high mask-wearing practice. Among men, current users were associated with non-compliance with mask-wearing compared to never users. Former users in both men and women were associated with non-compliance with mask-wearing. A difference in not wearing a mask by the types of TNP was also observed. Among men, cigarette users, compared to NCTNP users, were associated with non-compliance with mask-wearing. Meanwhile, there were negative associations between non-compliance with mask-wearing when the use decreased in men and increased in women. Associations between attempt to quit and not wearing a mask were not significant in both men and women.

The low compliance with mask-wearing of current users is similar to the result of the study in Hong Kong [14]. TNP use in situations where it was difficult to keep distance and inappropriate mask-wearing before and after the use may have affected the study results. The risk-avoidance tendency is related to mask-wearing, and smokers have a low risk-avoidance tendency [27], which can be inferred as the cause of non-compliance with mask-wearing. It is noteworthy that current and former users had a high non-compliance with mask-wearing than never users. In addition, the high association between cigarette users and not wearing a mask compared to NCTNP users can be interpreted considering that NCTNP users have fewer spatial restrictions and choose a smoking place with no other people easily [28,29]. Women had no significance due to the low frequency generally, however, a significant result was observed in the change of TNP use. Non-compliance with mask-wearing among those who increased TNP use was low. On the contrary, men wore a mask well when their use decreased. This may be due to differences in smoking areas between women and men. Women usually smoke in toilets or balconies at home, whereas men smoke in smoking rooms outside buildings and on the street [30]. Men smoke in public rather than in their own private spaces, so they would have been more likely to wear a mask as TNP use decreased. Moreover, since women are more concerned about their infection with COVID-19 [31], women would have complied all the better with wearing a mask in spite of the increase in TNP use. Attempt to quit did not show a significant difference in non-compliance with mask-wearing.

Smokers are more associated with COVID-19 morbidity than non-smokers [32,33]. Even though there was a study [22] reports that a significant difference in the morbidity of COVID-19 between smokers and non-smokers was absent, the results of our study cannot be excluded. This is because non-compliance with the mask-wearing of smokers can potentially transmit the virus to both smokers and non-smokers. TNP users were associated with non-compliance with mask-wearing and had a higher risk of developing severe COVID-19 infection conditions [34]. Moreover, if TNPs are used when gathering as a group or when social distancing is not practiced, an unpredictable spread can also occur to non-users and other users. Therefore, based on the results of this study, we suggest that more attention should be paid to the prevention of TNP use and cessation support during the epidemic of respiratory infectious diseases. In addition, it is necessary to identify and supplement cigarette users’ weaknesses in complying with mask-wearing compared to NCTNP users. Moreover, in the case of men, the smoking rate is very high compared to women, and the reduction in TNP use is related to wearing a mask, so it is necessary to actively improve the factors related to inhibiting attempts to quit.

### Strengths and limitations

In Korea, where the rate of mask-wearing was very high in the early stages of the COVID-19 pandemic, differences in non-compliance with mask-wearing were observed depending on the TNP use behaviors. This study has the following strengths.

First, studies on COVID-19 and smoking in Korea were mostly related to biological mechanisms. However, this study explored the correlation between TNP use behaviors and not wearing a mask and analyzed users’ behaviors in detail using data from a national-level project. In addition to existing studies that TNP users are at higher risk of contracting COVID-19, this study found that TNP use behaviors were associated with not wearing a mask, a risk factor for COVID-19 infection.

Second, the current TNP users were classified into cigarette users and NCTNP users, who have different use behaviors, and the difference in non-compliance with mask-wearing was confirmed. This analysis was not well addressed in existing studies in other countries, and this study was a new attempt focusing on restrictions on smoking areas depending on the products used.

### However, this study has several limitations.

First, this study was conducted using large-scale survey data in Korea, where mask-wearing is very high, and the odds of not wearing a mask differed by about 1.3 to 2.0 times. Still, the difference is about 1% in absolute values. However, 1% of the total population cannot be overlooked, and if it is possible to prevent serious infectious diseases such as COVID-19, an effect of more than 1% can be expected. In addition, the survey of this study was conducted in the early stages of the COVID-19 outbreak, and the significance of the study results will likely be more prominent when awareness is eased, and mask-wearing compliance is lowered.

Second, since a difference in the smoking rate between men and women exist, the analysis was stratified according to gender. However, significant results were not obtained in various variables because of the low percentage of women users.

Finally, when the changes in TNP use were asked in the 2020 Community Health Survey, the question was, ‘What are the changes compared to before the COVID-19 pandemic?’ It was not explored whether the changes were about quantity or frequency. In this study, we assumed that the participants would respond considering the amount and frequency of use overall.

Therefore, to obtain more meaningful results on the relationship between smoking behavior and mask-wearing, a comparative study supplementing the above limitations needs to be conducted in countries with low mask-wearing. Moreover, further analysis with follow-up data after 2020 will reveal differences according to the stage of the epidemic.

## Figures and Tables

**Figure f2-epih-44-e2022087:**
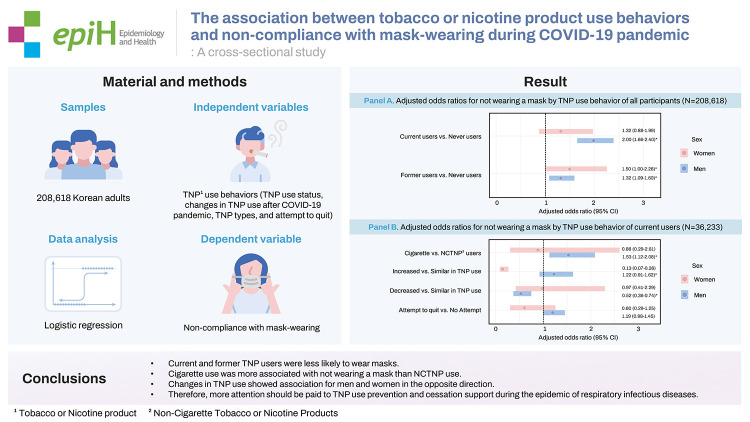


**Table 1.** Non-compliance with mask-wearing by socio-demographic characteristics of all participants (n=208,618)

Characteristics	Men			Women		
	Total	Non-compliance	p-value	Total	Non-compliance	p-value
	n (%)	n (weighted%)^1^		n (%)	n (weighted%)^1^	
Total	94,850 (45.47)	1,857 (1.27)		113,768 (54.53)	1,491 (0.74)	
Age (yr)			<0.001			<0.001
19-29	11,805 (12.45)	101 (0.80)		12,630 (11.10)	66 (0.50)	
30-39	11,308 (11.92)	108 (0.80)		12,340 (10.85)	77 (0.62)	
40-49	15,846 (16.71)	283 (1.28)		17,815 (15.66)	119 (0.50)	
50-59	18,951 (19.98)	432 (1.62)		22,253 (19.56)	216 (0.70)	
60-69	18,580 (19.59)	469 (1.69)		22,337 (19.63)	276 (0.69)	
≥70	18,360 (19.36)	464 (1.53)		26,393 (23.20)	737 (1.57)	
Occupations			<0.001			<0.001
Managers and professionals	11,106 (11.71)	132 (1.01)		10,784 (9.48)	48 (0.49)	
Clerks	9,498 (10.01)	109 (0.87)		9,277 (8.15)	54 (0.59)	
Service and sales workers	9,810 (10.34)	138 (1.21)		17,435 (15.33)	148 (0.69)	
Skilled agricultural and fishery workers	11,305 (11.92)	534 (4.56)		8,622 (7.58)	290 (2.89)	
Craft·elementary workers	26,299 (27.73)	506 (1.45)		13,904 (12.22)	209 (0.71)	
Unemployed	26,832 (28.29)	438 (1.02)		53,746 (47.24)	742 (0.77)	
No. of household members			0.001			<0.001
Single person	11,975 (12.63)	294 (1.63)		19,954 (17.54)	519 (1.44)	
Multi-person	82,875 (87.37)	1,563 (1.22)		93,814 (82.46)	972 (0.64)	
Education level			<0.001			<0.001
<Middle school	23,810 (25.10)	777 (2.27)		45,032 (39.58)	1,046 (1.33)	
High school	36,430 (38.41)	657 (1.28)		35,918 (31.57)	245 (0.52)	
≥College	34,610 (36.49)	423 (0.95)		32,818 (28.85)	200 (0.57)	
Area			<0.001			<0.001
Urban	54,272 (57.22)	630 (1.04)		65,169 (57.28)	391 (0.55)	
Rural	40,578 (42.78)	1,227 (2.23)		48,599 (42.72)	1,100 (1.62)	
Marital status			<0.001			<0.001
Married	66,590 (70.21)	1,380 (1.33)		71,185 (62.57)	751 (0.64)	
Single	28,260 (29.79)	477 (1.16)		42,583 (37.43)	740 (0.91)	
Monthly household income (million Korea won)			<0.001			<0.001
<2	25,597 (26.99)	671 (1.69)		38,669 (33.99)	867 (1.27)	
2-4	29,836 (31.46)	599 (1.31)		32,013 (28.14)	330 (0.59)	
>4	39,417 (41.56)	587 (1.09)		43,086 (37.87)	294 (0.58)	

1 Except for total non-compliance (indicated by not weighted %).

**Table 2.** Non-compliance with mask-wearing by TNP use behaviors of all participants (n=208,618)

Characteristics	Men			Women		
	Total	Non-compliance	p-value	Total	Non-compliance	p-value
	n (%)	n (weighted%)^1^		n (%)	n (weighted%)^1^	
Total	94,850 (45.47)	1,857 (1.27)		113,768 (54.53)	1,491 (0.74)	
TNP use status			<0.001			<0.001
Current users						
Cigarette users	28,831 (30.40)	751 (1.86)		2,618 (2.30)	33 (0.87)	
NCTNP users						
HTPs only	1,472 (1.55)	16 (0.81)		121 (0.11)	1 (0.86)	
ECs only	482 (0.51)	1 (0.02)		59 (0.05)	1 (0.66)	
Dual use of CCs & HTPs	1,377 (1.45)	25 (1.18)		68 (0.06)	1 (0.84)	
Dual use of CCs & ECs	609 (0.64)	6 (0.65)		129 (0.11)	1 (0.68)	
Dual use of HTPs & ECs	115 (0.12)	2 (0.56)		12 (0.01)	0 (0.00)	
Triple use	309 (0.33)	7 (2.12)		31 (0.03)	1 (2.75)	
Former users^2^	34,315 (36.18)	697 (1.26)		3,157 (2.77)	43 (0.93)	
Never users^3^	27,340 (28.82)	352 (0.78)		107,573 (94.55)	1,410 (0.73)	
Changes in TNP use after the COVID-19 outbreak			<0.001			0.149
Increased	3,198 (3.37)	86 (1.99)		386 (0.34)	2 (0.14)	
Similar	25,739 (27.14)	654 (1.77)		2,159 (1.90)	30 (1.00)	
Decreased	4,258 (4.49)	68 (0.92)		493 (0.43)	6 (0.96)	
Not applicable	61,655 (65.00)	1,049 (1.03)		110,730 (97.33)	1,453 (0.73)	
Attempt to quit			<0.001			0.355
Yes	15,007 (15.82)	313 (1.49)		1,489 (1.31)	17 (1.05)	
No	18,188 (19.18)	495 (1.87)		1,549 (1.36)	21 (0.69)	
Not applicable	61,655 (65.00)	1,049 (1.03)		110,730 (97.33)	1,453 (0.73)	

TNP, tobacco or nicotine product; HTP, heating tobacco products; EC, electronic cigarettes; CC, combustible cigarettes=cigarettes; NCTNP, non-cigarette tobacco or nicotine product; COVID-19, coronavirus disease 2019.

1 Except for total non-compliance (indicated by not weighted %).

2 Former use of any TNPs.

3 Never use any TNPs.

## SUPPLEMENTARY MATERIALS

Korean version is available at https://www.e-epih.org/.

Supplementary Material 1. Adjusted odds ratio(aOR) for not wearing a mask of all participants (N=208,618)

Supplementary Material 2 Adjusted odds ratio(aOR) for not wearing a mask of current users (N=36,233)
