# Supplementary material for: The association between tobacco or nicotine product use behaviors and non-compliance with mask-wearing during the COVID-19 pandemic: a cross-sectional study in Korea
Source: Epidemiol Health. 2022 Oct 7;44:e2022087. doi: 10.4178/epih.e2022087 (PMC10089704; doi:10.4178/epih.e2022087)
Supplement: Supplementary file 1 [file epih-44-e2022087-Korean-Supplementary.docx]

**The association between tobacco or nicotine product use behaviors and**

**non-compliance with mask-wearing during COVID–19 pandemic: a cross-sectional study**

**Da-eun Lee^1, 2^, Heewon Kang^3^, Sung-il Cho^1, 3^**

**^1^ Department of Public Health Science, Graduate School of Public Health, Seoul National University, Seoul, Korea; ^2^Medical Intensive Care Unit, Seoul ST. Mary's Hospital, The Catholic University of Korea, Seoul, Korea; ^3^Institute of Health and Environment, Seoul National University, Seoul, Korea**

**Corresponding author: Sung-il CHO**

**Department of Public Health Science, Graduate School of Public Health, and Institute of Health and Environment, Seoul National University**

**1 Gwanak-ro, Gwanak-gu, Seoul 08826, Republic of Korea**

**Email: persontime@hotmail.com**

**ORCID:0000-0003-4085-1494**

**ABSTRACT**

OBJECTIVES: It is necessary to investigate TNP (Tobacco or Nicotine Product) use which acts as a risk factor for COVID-19 infection. Especially, wearing a mask is difficult to practice while using TNP. Therefore, this study aimed to examine the association between TNP use behaviors and non-compliance with mask-wearing during the COVID-19 pandemic.

METHODS: The Samples of 208,618 Korean adults from 2020 Community Health Survey in Korea were used. As an independent variable, TNP use behaviors such as TNP use status, changes in TNP use after COVID-19, TNP types, and attempt to quit were analyzed. Logistic regression was performed on sex-stratified participants.

RESULTS: Among men, the Odds ratio (OR) of current TNP and former TNP users were 2.00 [95%CI (1.66, 2.40)] and 1.32 [95%CI (1.09, 1.60)], respectively, compared to never users. In women OR was 1.50 [95%CI (1.00-2.26)] for former users. Cigarette use was more associated with not wearing a mask than NCTNP(Non-Cigarette Tobacco or Nicotine Products) use [OR=1.53, 95%CI (1.12, 2.08)]. Men whose TNP use decreased had lower non-compliance [OR=0.52, 95%CI (0.36, 0.74)]; while women whose TNP use increased had lower non-compliance [OR= 0.13, 95%CI (0.07, 0.26)].

CONCLUSIONS: Current and former users were less likely to wear masks. Cigarette use was more associated with not wearing a mask than NCTNP use. Changes in TNP use showed association for men and women; however, in the opposite direction. Therefore, more attention should be paid to TNP use prevention and cessation support during the epidemic of respiratory infectious diseases. Moreover, it is necessary to identify risk factors of cigarette users in compliance with mask-wearing.

**KEY WORDS:** TNP use behaviors, Non-compliance with mask wearing, COVID-19, Respiratory infectious disease, Infection prevention, TNP using prevention

**INTRODUCTION**

흡연은 각종 암과 관상동맥질환, 심혈관계 질환, 죽상경화성 말초혈관질환 등에 큰 위험요소로 작용하며, 만성폐쇄성폐질환에는 가장 중요한 위험인자이다[1]. 이로 인해 전 세계적으로 흡연으로 인한 사망자 수가 연간 8백만 명 이상에 이르고 경제적 부담이 상당하다[2].

국제개발협력기구(Organization for Economic Cooperation and Development)에 따르면 2019년 매일 흡연자(만 15세 이상)의 OECD 평균 비율은 16.5%이고 한국은 16.4%로 38개국 중 21위이다[3]. 한국의 남성 매일 흡연자의 비율은 28.5%로 OECD 평균(20.6%)을 훨씬 상회하는데, 43개국 중에서 9번째로 높은 순위이다[4]. 국내를 비롯하여 전세계적으로 남성의 흡연(32.6%)은 여성 흡연(6.5%)대비 매우 높은 수준으로, 주목되고 있다[5].

흡연의 심각성에 전 지구적 대응으로 WHO(World Health Organization)는 2005년 2월 27일 국제 협약인 ‘담배규제기본협약(FCTC: Framework Convention on Tobacco Control)’을 발효하여 추진했다. 전 세계 인구의 90% 이상이 담배 규제 정책을 적용 받고 있으며[6] 우리나라는 2005년 5월에 비준하여 국제 협약을 따르고 있다. 국가 자체적으로도 국민건강증진법을 제정하여 법적 근거를 토대로 정책을 마련하고 각종 사업과 연구 등을 진행하고 있다.

2019년 12월 발발한 코로나바이러스감염증-19(COVID-19, 이하 코로나19)로 인해 금연의 중요성은 더 대두되고 있다. 코로나19가 비말을 통해 전파되고, 심한 폐렴을 불러오기 때문에 취약한 위생 습관을 가졌으며 기존에 폐 기능이 좋지 않은 흡연자들은 코로나19에 감염될 위험이 더 높고 악화되기 쉽다[7,8]. 또한 흡연 중 오염된 손과 담배는 입과 접촉하게 되어, 코로나19 전파의 기회가 된다[7]. 흡연은 연기를 내뱉게 하고, 기침과 재채기로 코로나19 바이러스(SARS-CoV-2)가 포함된 비말을 발생시킬 수 있어 주변 흡연자 뿐만 아니라 간접 흡연자에게도 코로나19 전파 경로로 작용한다[7]. 그러므로 공중 보건을 위해 코로나19 범유행(COVID–19 pandemic, 이하 코로나19 유행) 상황에서는 금연의 중요성을 다뤄야 한다[9].

코로나19 바이러스 전파 차단을 위한 사회적 거리두기와 마스크 착용의 중요성은 이미 여러 연구를 통해 밝혀져 있다. 마스크가 코로나19 바이러스의 전파 경로를 차단시키기 때문에 착용률이 높을수록 코로나19 감염이 줄어들었다[10]. 사회적 거리두기 또한 코로나19 감염 수를 낮추고 코로나19 유행 상황을 늦추었다[11]. 우리나라에서는 실내 전체 및 실외에서 2m 이상 거리 유지가 되지 않는 경우 마스크 착용을 의무화하고 있다. 다만 흡연 시에는 예외 상황으로 거리두기를 지킬 것과 흡연 전후에 마스크를 착용하도록 규정을 두고 있다[12]. 그러나 마스크 착용률이 높은 홍콩[13]에서도 흡연자의 32.4%가 흡연 직후에 마스크를 착용하지 않았고 74.3%의 흡연자들은 흡연 중 거리두기를 지키지 않았다[14]. 즉 흡연 시와 전후에 주변 흡연자와 비흡연자에게 바이러스 전파 위험이 있을 것으로 예상할 수 있다. 이에 흡연율이 높은 국내에서도 코로나19 유행 기간 TNP(Tobacco or Nicotine Products) 사용자들의 마스크 미착용에 대해 파악할 필요가 있다. 그에 따라 호흡기 감염병 유행이 반복되는 상황에서 TNP 사용 예방 및 금연 지원 활동의 중요성을 더욱 제고하고 방역수칙을 개선하기 위해 코로나19 유행 상황에서 TNP 사용 행태와 마스크 미착용의 연관성을 분석하고자 한다.

**MATERIALS AND METHODS**

**Study population**

본 연구는 질병관리청 지역사회건강조사 2020년 원시자료를 이용한 2차 분석 연구이다. 2020년 8월 16일부터 2020년 10월 31일까지 표본가구에 거주하는 만 19세 이상 성인을 대상으로, 훈련된 조사원이 1:1 전자설문조사를 통해 자료를 수집했다. 229,269건의 조사 결과 중 본 연구에서 변수로 고려한 문항에 ‘모름’, ‘응답거부’, ‘항목 무응답’ 등의 결측치를 제외한 208,618명의 데이터를 분석에 이용하였다.

**Measures**

***Tobacco/nicotine product use behaviors***

TNP 사용 행태에는 TNP 사용 상태, TNP 사용 변화, TNP 종류, 금연 시도 여부가 포함되었다. TNP 현재 사용 여부는 ‘현재’ 또는 ‘최근 1달동안’을 기준으로 판단되었고, 현재는 사용하지 않지만 과거에 사용 경험이 있을 경우 과거사용자, 평생 사용 경험이 없는 경우 비사용자로 분류되었다. TNP 사용 변화는 코로나19 유행 이전과 비교하여 증감을 확인하였고 코로나19 유행 전부터 사용하지 않은 경우는 ‘해당없음’으로 처리했다. TNP에는 일반담배(궐련), 궐련형 전자담배, 액상형 전자담배가 포함되었으며 그 외 머금는 담배(스누스), 물담배(waterpipe tobacco 또는 shisha 또는 hookah), 시가 등의 기타담배는 2020년에 설문이 이루어지지 않아 본 연구에서도 제외하였다. 금연 시도 여부는 현재 사용자에게 최근 1년을 준거기간으로, 하루 이상 금연한 적 있는지를 질문하였다.

***Tobacco/Nicotine product use status***

담배 시장의 빠른 변화로 갖가지 형태, 맛, 디자인, 사용 방식을 갖춘 담배제품들이 출시되었고, 이에 따라 담배를 지칭하고 분류하는 기준 또한 여러 방식으로 이용되었다. 전통적 담배와 비전통적 담배, 또는 태우는 담배와 연기 없는 담배 등 연구마다 분류하는 방법과 정의가 상이하다~~.~~

본 연구에서는 사용하는 담배 또는 니코틴 제품별로 전체적인 빈도를 살펴보기 위해 사용자를 7개의 그룹, 즉 궐련(일반담배) 단독 사용자, 궐련형 전자담배 단독 사용자, 액상형 전자담배 단독 사용자, 궐련과 궐련형 전자담배 이중 사용자, 궐련과 액상형 전자담배 이중 사용자, 궐련형 및 액상형 전자담배 이중사용자, 삼중 사용자로 나누어 비교하였다. 전체 참가자를 대상으로 한 회귀분석에서는 담배 또는 니코틴 제품(Tobacco or Nicotine Products, TNP) 사용 여부에 따른 비교를 위해 현재사용자, 과거사용자, 비사용자로 구분하였다. 이 중 현재사용자를 대상으로 한 회귀분석에서는 흡연 행태의 차이에서 오는 마스크 미착용의 비교를 위해 현재사용자를 ‘일반담배(궐련, Cigarettes) 사용자’와 ‘비궐련 담배 또는 니코틴 제품(Non-Cigarette Tobacco or Nicotine Products, NCTNPs) 사용자’로 분류하였다. NCTNPs 사용자에는 궐련형 전자담배 및 액상형 전자담배 사용자가 속하며, 일반담배와 액상형 전자담배 또는 궐련형 전자담배를 병용하는 사용자도 포함되었다.

***Mask wearing***

마스크 착용은 실내와 실외를 구분하여 조사되었고, 실내의 경우 ‘불특정 다수가 이용하는 실내시설(예시 생략)에서 마스크를 착용하셨습니까?’로 질문하였다. 실외 마스크 착용은 ‘야외에서 사람 간 2m이상 거리두기가 어려운 경우 마스크를 착용하셨습니까?’로 질문하였고, ‘매우 그렇다’, ‘그렇다’를 착용, ‘그렇지 않다’를 미착용으로 분류하였다. 본 연구에서는 실내와 실외에서 모두 마스크를 착용한 경우를 마스크 착용으로 분류하였고 실내 또는 실외 둘 중 하나라도 마스크 착용이 안 된 경우 미착용으로 분류하였다.

***Other covariates***

모든 분석은 성별로 층화되었으며 보정된 인구통계학적 특성 변수는 나이, 직업, 가구원 수, 교육 수준, 거주 지역, 혼인상태, 월 가구 소득이다. 각 변수들은 선행연구에서 노출변수 및 설명변수와의 연관성을 확인하였다.

2020년 한국 만 15세 이상 남성 매일 흡연자는 27.8%, 여성 매일 흡연자는 3.9%로 성별에서 큰 차이를 보이고[15] 남녀 흡연 차이의 주요한 원인은 흡연 행태의 차이다[16]. 직업과 교육수준, 소득 또한 흡연 행태와 연관성이 있으며[17], 이혼하거나 미혼인 사람이 금연을 할 가능성이 적었다[18].

여성은 남성보다 마스크를 더 잘 착용했으며 연령이 감소함에 따라 마스크 착용 오즈비도 감소했다[19]. 도시와 교외는 마스크 착용률이 비슷하지만 지방에서는 착용률이 현저히 낮았다[19]. 일을 하는 사람은 무직에 비해, 그리고 집에서 일할 때 보다 집 밖에서 일을 할 때 마스크 미착용과 연관성이 있었다[20]. 같은 연구에서 낮은 교육 수준 또한 마스크 미착용과 관련이 있었고, 고소득자와 기혼 그룹은 더 마스크를 잘 착용했다[21].

**Statistical analysis**

표본이 복합표본설계(complex sampling design) 하에서 추출되어, 평균 및 분산 추정 시 모집단을 대표할 수 있도록 가중치, 층화변수, 집락변수를 고려하여 산출하였다. 통계 분석은 유의 수준 p<0.05로 설정하여 SAS 9.4 version(surveylogistic procedure)을 이용하였다. 로지스틱 회귀분석은 먼저 전체 참가자를 대상으로 TNP 사용 상태에 따른 마스크 미착용을 분석하였고, TNP 종류, TNP 사용 변화, 금연 시도에 따른 마스크 미착용은 현재 사용자를 대상으로 진행되었다. 또한 성별에 따라 흡연률에 차이가 있어 남녀로 층화하여 분석하였다. 나이 변수는 linear한 형태를 확인한 후 연속형 변수로 사용하였다. 각 회귀분석에서 도출된 OR(odds ratio)는 마스크 착용 odds에 대한 마스크 미착용의 odds를 나타낸다.

이외에도 VIF(분산팽창요인, Variance Inflation Factors) 계산을 통해 독립변수 간의 다중공선성(Multicollinearity)을 배제하였다. 또한 모델의 적합도 검증을 위해 AIC(Akaike information criterion)를 비교하였으며 2가지 로지스틱 회귀분석 모두 가장 낮은 AIC를 보인 모델을 사용하였다.

**Ethical statement**

본 연구는 서울대학교 생명윤리심의위원회에서 2022년 5월 6일 심의 면제를 받았다(IRB No. E2205/002-005).

**RESULTS**

전체 참가자의 인구통계학적 특성별 마스크 미착용률이 표 1에 제시되어 있다. 전체 남성의 1.27%, 전체 여성의 0.74%가 마스크를 착용하지 않아 높은 수준의 마스크 착용 실천을 확인할 수 있다. 다만 여성 대비 남성의 마스크 미착용률은 1.72배 가량 높아 성별의 차이를 볼 수 있다. 남성에서 마스크 미착용률은 연령이 높아질수록 대체적으로 증가했으며 여성은 70세 이상의 마스크 미착용률이 1.57%로 다른 연령대에 비해 높았다. 직업 군 비교에서는 농림어업종사자의 마스크 미착용률이 남녀 모두 가장 높았고 1인가구는 1인가구가 아닌 집단보다 마스크 미착용률이 높았다. 교육수준은 대체적으로 낮을수록, 도시보다 지방 거주자의 마스크 미착용률이 높았다. 남성에서는 기혼자, 여성에서는 기혼이 아닌 사람이 마스크 미착용률이 높아 상반된 결과를 보였고 소득수준은 공통적으로 낮을수록 마스크 미착용률이 높았다.

| (표1) |
| --- |

표 2에는 TNP 사용 행태별 마스크 미착용률이 성별로 구분되어 나타나 있다. TNP 사용 상태에서 남성은 과거사용자(36.18%), 여성은 비사용자 비율(94.55%)이 가장 높았으며 마스크 미착용률은 남녀 모두 삼중 사용자에서 가장 높았다. 코로나19 유행 이전 대비 TNP 사용의 변화는 남녀 모두 증가한 사람보다 감소한 사람이 조금 더 많았다. TNP사용이 증가한 남성은 사용이 감소한 남성보다 마스크 미착용률이 높았다. 반면 금연시도는 하지 않은 그룹이 시도한 그룹보다 남녀 모두 조금 더 많았다. 금연시도를 하지 않은 남성은 금연시도를 한 남성보다 마스크 미착용률이 높았다.

| (표2) |
| --- |

마스크 미착용에 대한 로지스틱 회귀분석 결과는 그림1 에서 확인할 수 있다. 전체 참가자의 담배 또는 니코틴 제품 사용 상태에 따른 마스크 미착용을 살펴보면(Panel A), 남성에서 주로 유의성이 나타났다. 남성 현재 사용자의 마스크 미착용 odds는 남성 비사용자 대비 2.00배 높았다(OR=2.00, 95%CI [1.66, 2.40]). 남성 과거사용자도 비사용자보다 마스크 미착용 odds가 1.32배 높았으며(OR=1.32, 95%CI [1.09, 1.60]) 여성 과거사용자의 경우 1.50배 컸다(OR=1.50, 95%CI [1.00-2.26]). 현재 사용자 내에서 TNP 사용 행태에 따른 마스크 미착용을 비교해보면(Panel B), 남성 NCTNP 사용자 대비 남성 일반담배 사용자가 마스크 미착용과 연관이 있었다(OR=1.53, 95% CI [1.12, 2.08]). 여성에서는 두 그룹간의 유의한 차이가 없었다. 코로나19 유행 이전 대비 사용 변화는 남녀에서 상이한 결과를 보였다. 남성에서는 사용이 감소할 때 마스크 미착용과 음의 연관성을 보인 반면(OR=0.52, [0.36, 0.74]) 여성에서는 사용이 증가할 때 마스크 미착용과 음의 연관성을 보였다(OR= 0.13, 95%CI [0.07, 0.26]). 금연시도에서는 남녀 모두 유의성이 없었다. 인구통계학적 변수에 따른 마스크 미착용은 부록으로 첨부하였다(supplement 1, 2)

| (그림1) |
| --- |

**DISCUSSION**

**MAIN FINDINGS**

본 연구에서는 한국의 코로나19 유행 시기 TNP 사용 행태와 마스크 미착용에 대해 알아보았다.

코로나19 유행 이전 대비 TNP 사용의 변화는 증가한 사람보다 감소한 사람이 조금 더 많았다. 이는 흡연의 증가가 더 많았다는 네덜란드나 영국의 연구[22,23]와는 상반되지만, 이탈리아의 연구[24]와는 비슷한 결과이다. 코로나19는 TNP 사용의 증감에 모두 영향을 주는 것으로 보인다.

국내 마스크 착용률은 남성 98.73%(미착용 1.27%), 여성 99.26%(미착용 0.74%)이었다. 이는 비슷한 시기 북유럽의 마스크 착용률(15% 이하)과 [16], 유럽에서 높은 편이라고 알려진 스페인(96.4%), 이탈리아(93.9%)에 비해서도[25] 매우 높은 수준이다. 28개국 간의 비교에서 한국의 마스크 착용률은 1위였는데[26], 여러 문헌들에서 그 원인을 집단주의와 이전 마스크 착용 경험 등으로 설명하고 있다[27].

하지만 이렇듯 높은 마스크 착용 실천 상황에서도 TNP 사용 행태 별로 미착용에 대한 차이가 있었다. 비사용자와 비교했을 때 현재 사용자는 남성에서 마스크 미착용과 연관성이 있었고 과거사용자는 남녀 모두에서 미착용과 연관성이 있었다. 담배 또는 니코틴 제품에 따라서도 차이가 있었는데, 남성에서 NCTNPs 대비 일반담배 사용이 마스크 미착용과 연관성이 있었다. 또한 남성은 사용이 감소할 때, 여성에서는 사용이 증가할 때 마스크 미착용과 음의 연관성이 있었다. 금연시도는 남녀 모두 연관성이 없었다.

현재사용자의 낮은 마스크 착용 실천은 홍콩의 연구[14]와 유사한 결과이다. 거리두기가 어려운 상황에서의 TNP 사용과 사용 전후의 미흡한 마스크 착용이 결과에 영향을 미쳤을 수 있다. 위험 회피 성향은 마스크 착용과 관련이 있는데, 흡연자는 위험 회피 성향이 낮아[28] 미착용에 대한 원인으로 추측해 볼 수 있다. 현재사용자뿐만 아니라 과거사용자도 비사용자 대비 마스크 미착용이 높아 주목할 만하다. 또한 일반담배 사용자가 NCTNPs 사용자에 비해 마스크 미착용 연관성이 높은 것은 NCTNPs 사용이 공간적 제약이 적어 타인이 없는 흡연 장소를 선택하기 용이하다는 점[29,30]에서 결과를 해석할 수 있다. 대체적으로 여성은 빈도수가 적어 유의성이 없었으나 TNP 사용 변화에서는 유의한 결과가 있었다. 사용이 증가한 사람의 마스크 미착용이 오히려 낮았는데 남성에서는 사용이 감소할 때 마스크 미착용이 낮은 것과 상반된 결과이다. 이는 여성과 남성의 흡연 장소에 따른 차이점으로 원인을 생각해볼 수 있다. 여성은 주로 가정 실내 화장실, 가정 실내 베란다 등에서, 남성은 건물 외 흡연실, 길거리에서 흡연하는 경우가 유의하게 높다[31]. 개인적인 공간보다 다수의 타인이 있는 곳에서 흡연하는 남성은 TNP 사용이 감소하면서 마스크를 더 잘 착용했을 것이다. 또한 여성은 코로나19 감염에 대한 염려가 크기 때문에[32] 여성의 TNP 사용 증가는 오히려 마스크 착용에 기인했을 가능성이 있다. 금연 시도는 마스크 미착용에 유의한 차이를 보이지 않았다.

흡연자는 비흡연자 대비 코로나19 이환과 관련이 있다[33,34]. 흡연자와 비흡연자 간의 코로나19 이환에 큰 차이가 없다는 연구[35]도 있으나 흡연자의 마스크 미착용은 흡연자와 비흡연자 모두에게 바이러스 전파 가능성이 있어 코로나19 이환의 동질성으로 본 연구의 결과를 배제할 수 없다. TNP 사용자는 마스크 미착용과 연관성이 있으며 코로나19 이환시 중증으로 진행될 위험이 더욱 높다[36]. 또한 그룹으로 모이거나 타인과 거리두기가 지켜지지 않을 때도 TNP를 사용한다면 비사용자와 다른 사용자에게도 전파 경로를 예측할 수 없는 확산이 일어날 수 있다. 따라서 본 연구의 결과를 토대로 호흡기 감염병 유행 시기에는 더욱 TNP 사용 예방과 금연 지원에 관심을 가질 것을 제안한다. 또한 NCTNPs 대비 일반담배 사용자가 마스크 착용을 준수하는데 있어 취약한 점들을 파악하여 보완할 필요가 있다. 특히 남성의 경우 여성 대비 흡연율이 매우 높고 흡연의 감소가 마스크 착용과 관련이 있어, 금연의 저해 요인을 적극적으로 개선시킬 필요가 있다.

**STRENGTHS AND LIMITATIONS**

코로나19 유행 초기 마스크 착용률이 매우 높은 국내에서도 TNP 사용 행태에 따라서 마스크 미착용에 차이가 있음을 파악하였다. 본 연구 결과는 다음과 같은 강점을 갖는다.

첫째, 현재 국내에서 코로나19와 흡연에 대한 연구는 생물학적 기전에 관련된 것이 대부분인데TNP 사용 행태와 마스크 착용과의 상관성을 새롭게 탐구하였으며 국가 단위 사업의 데이터를 이용하여 사용자들의 행태를 세분화하여 분석할 수 있었다. 흡연자의 코로나19 감염이 더 위험하다는 기존 연구들에 추가로, 코로나19 감염의 위험 요인인 마스크 미착용이 TNP 사용 행태에 따라 연관성이 있음을 밝혔다.

둘째, TNP 현재 사용자를 사용 행태에 차이가 있는 일반담배 사용자와 NCTNPs 사용자로 구분하여 마스크 미착용의 차이를 확인했다. 이는 기존 해외 연구에서도 잘 다뤄지지 않은 분석이며 사용 제품에 따라 장소적 제약 등이 다름에 착안해 진행한 새로운 시도였음을 밝힌다.

하지만 이 연구는 몇 가지 한계점을 갖는다.

첫째, 본 연구는 마스크 착용이 매우 높은 한국에서 대규모 조사자를 대상으로 진행되어 마스크 미착용 OR(Odds Ratio)이 행태별로 1.3~2배 가량 차이가 났지만 절대값에서는 1% 가량의 차이임을 유의해야한다. 하지만 전체 인구집단에서 1%는 간과할 수 없는 비중이며 코로나19와 같이 중증으로 진행될 수 있는 감염병을 예방할 수 있다면 1% 이상의 효과를 기대할 수 있을 것이다. 또한 본 연구의 조사는 코로나19 유행 초기에 이루어졌는데 경각심이 완화되고 마스크 착용 준수가 낮아지는 시점에서는 연구 결과의 의미가 더 클 것으로 예상한다.

둘째로, 남녀의 흡연율에 차이가 크므로 성별을 층화하여 분석했지만, 여성은 사용자 비율이 낮아 여러 변수에서 유의한 결과를 얻지 못하였다.

마지막으로, 지역사회건강조사에서 TNP 사용의 변화를 설문할 당시 ‘코로나19 유행 이전과 비교했을 때 어떤 변화가 있습니까?’라고 조사하여 응답이 양이나 빈도 중 어떤 것에 관한 것인지 정확히 파악되지 않았다. 본 연구에서는 조사 대상자가 사용량과 사용의 빈도를 전반적으로 고려하여 응답했을 것으로 가정하고 진행되었다.

따라서, 위와 같은 한계점을 보완하고 마스크 착용이 낮은 국가와 비교 연구를 진행한다면 흡연 행태와 마스크 착용의 연관성에 대한 더 의미 있는 결과를 도출할 수 있을 것이다. 또한 2020년 이후의 후속 자료로 추가 분석 시 유행의 진행 단계에 따른 차이를 밝힐 수 있을 것이다.

**REFERENCES**

1. Sherman CB. Health effects of cigarette smoking. Clin Chest Med. 1991 Dec;12(4):643-658. Doi: [10.1016/S0272-5231(21)00814-5](https://doi.org/10.1016/S0272-5231(21)00814-5)
2. WHO. Tobacco [Internet]. Geneve: WHO. 2022 May 24[updated 2022 May 24; cited 2022 May 26]. Available from: <https://www.who.int/news-room/fact-sheets/detail/tobacco>
3. Daily smokers(indicator) [Internet]. Paris: OECD. [date unknown; cited 2022 May 26]. OECD (2022), Doi: 10.1787/1ff488c2-en
4. Non-Medical Determinants of Health  : Tobacco consumption [Internet]. Paris: OECD. c2000-2021 [cited 2022 May 26]; Available from: <https://stats.oecd.org/Index.aspx?DataSetCode=HEALTH_STAT>
5. Dai X, Gakidou E, Lopez AD. Evolution of the global smoking epidemic over the past half century: strengthening the evidence base for policy action. Tobacco control. 2022 January; 31(2):129-137. Doi: 10.1136/tobaccocontrol-2021-056535
6. FCTC. WHO Framework Convention on Tobacco Control[Internet]. Washington: FCTC; n.d. [Retrieved 2022 May 26] Available from: <https://fctc.who.int/who-fctc/overview/parties>
7. Ahmed N, Maqsood A, Abduljabbar T, Vohra F. Tobacco Smoking a Potential Risk Factor in Transmission of COVID-19 Infection. Pakistan Journal of Medical Sciences. 2020 May 19; 36(COVID19-S4):104–107. Doi: [10.12669/pjms.36.COVID19-S4.2739](https://doi.org/10.12669/pjms.36.COVID19-S4.2739)
8. Palipudi K, Rizwan SA, Sinha DN, Andes LJ, Amarchand R, Krishnan A, et al. Prevalence and sociodemographic determinants of tobacco use in four countries of the World Health Organization: South-East Asia region: findings from the Global Adult Tobacco Survey. Indian J Cancer. 2014;51(5): 24-32. Doi: 10.4103/0019-509X.147446
9. Berlin I, Thomas D, Faou LA, Cornuz J. COVID-19 and Smoking. Nicotine & Tobacco Research. 2020 April 3;22(9):1650–1652. Doi: [10.1093/ntr/ntaa059](https://doi.org/10.1093/ntr/ntaa059)
10. Kazuyuki S, Hasegawa T, Kano N, Okamoto Y. A study of the effect of wearing face masks in preventing COVID-19 transmission in the United States of America. Public Administration and Policy: An Asia-Pacific Journal. 2021 Oct 26;24(3):275-289. Doi: 10.1108/PAP-08-2021-0046
11. Matrajt L, Leung T. Evaluating the Effectiveness of Social Distancing Interventions to Delay or Flatten the Epidemic Curve of Coronavirus Disease. Emerging infectious diseases. 2020 Aug;26(8):1740–1748. Doi: 10.3201/eid2608.201093
12. Handbook of orders to comply with quarantine guidelines for wearing masks and imposing fines for negligence(4^th^ -1 edition) “Korean, author’s translation” The Korea Disease Control and Prevention Agency. 2021 Nov 26. P.11&30.
13. Cheng VCC, Wong SC, Chuang VWM, So SYC, Chen JHK, Sridhar S, To KKW, Chan JFW, Hung IFN, Ho PL, Yuen KY. The role of community-wide wearing of face mask for control of coronavirus disease 2019 (COVID-19) epidemic due to SARS-CoV-2. Journal of Infection. 2020 July ;81(1):107-114. Doi:10.1016/j.jinf.2020.04.024.
14. Sun Y, Lam TH, Cheung Y, Wang MP, Wu Y, Chen J, et al. First Report on Smoking and Infection Control Behaviours at Outdoor Hotspots during the COVID-19 Pandemic: An Unobtrusive Observational Study. International journal of environmental research and public health. 2021 Jan 25;18(3):1031. Doi: [10.3390/ijerph18031031](https://doi.org/10.3390/ijerph18031031)
15. Non-Medical Determinants of Health  : Tobacco consumption [Internet]. Paris: OECD. c2000-2021 [cited 2022 September 1]; Available from: <https://stats.oecd.org/Index.aspx?DataSetCode=HEALTH_STAT>
16. Bauer [T](https://onlinelibrary.wiley.com/action/doSearch?ContribAuthorRaw=Bauer%2C+Thomas), [Göhlmann](https://onlinelibrary.wiley.com/action/doSearch?ContribAuthorRaw=G%C3%B6hlmann%2C+Silja) S, [Sinning](https://onlinelibrary.wiley.com/action/doSearch?ContribAuthorRaw=Sinning%2C+Mathias) M. Gender differences in smoking behavior. Helath Economics. 2007 July 09;16(9):895-909. Doi: 10.1002/hec.1259
17. Wang Q, Shen JJ, Sotero M, Li CA, Hou Z. Income, occupation and education: Are they related to smoking behaviors in China?. PLOS ONE. 2018 February 8;13(2): e0192571 [Doi: 10.1371/journal.pone.0192571](https://doi.org/10.1371/journal.pone.0192571)
18. Chandola T, Head J, Bartley M. Socio-demographic predictors of quitting smoking: how important are household factors?. Addiction. 2004 April 23;99(6):770-777. Doi: 10.1111/j.1360-0443.2004.00756.x
19. Haischer MH, Beilfuss R, Hart MR, Opielinski L, Wrucke D, Zirgaitis G. Who is wearing a mask? Gender-, age-, and location-related differences during the COVID-19 pandemic. PLoS ONE. 2020 Oct 15;15(10). Doi: [10.1371/journal.pone.0240785](https://doi.org/10.1371/journal.pone.0240785)
20. Goicoechea BE, Chang TH, Kim E, LaRocca S, Morris K, Deng X, et al. Global trends and predictors of face mask usage during the COVID-19 pandemic. BMC Public Health. 2021 Nov 15;21:2099. Doi: [10.1186/s12889-021-12175-9](https://doi.org/10.1186/s12889-021-12175-9)
21. Zhang L, Zhu S, Yao H, Li M, Si G, Tan X. Study on Factors of People's Wearing Masks Based on Two Online Surveys: Cross-Sectional Evidence from China. Int J Environ Res Public Health. 2021 Mar 26;18(7):3447. Doi: 10.3390/ijerph18073447
22. Bommele J, Hopman P, Walters BH, Geboers C, Croes E, Fong GT, et al. The double-edged relationship between COVID-19 stress and smoking: Implications for smoking cessation. Tobacco induced diseases. 2020 July;18. Doi: [10.18332/tid/125580](https://doi.org/10.18332/tid/125580)
23. Dimitra K, Aleksandra H, Olga P, Sarah EJ, Jamie B, Lion S. Associations between vaping and Covid-19: Cross-sectional findings from the HEBECO study. Drug and Alcohol Dependence. 2021 April 1;221. doi: [10.1016/j.drugalcdep.2021.108590](https://doi.org/10.1016/j.drugalcdep.2021.108590).
24. Caponnetto P, Inguscio L, Saitta C, Maglia M, Benfatto F, Polosa R. Smoking behavior and psychological dynamics during COVID-19 social distancing and stay-at-home policies: A survey. Health psychology research. 2020 May 27;8(1)). Doi: [10.4081/hpr.2020.9124](https://doi.org/10.4081/hpr.2020.9124)
25. How often have you worn a face mask outside your home to protect yourself or others from coronavirus (COVID-19)? [Internet]. Hamburg:Statista. 2020-2021. [updated 2021 Jan 10; cited 2022 May 26]. Available from: <https://www.statista.com/statistics/1114375/wearing-a-face-mask-outside-in-european-countries/>
26. Gallup International Association. The coronavirus: A vast scared majority around the world. Washington D.C.:Gallup International Association; 2020. 6p. [Cited 2020 May 26]. Available from: <https://www.gallup-international.com/survey-results/survey-result/the-coronavirus-a-vast-scared-majority-around-the-world>
27. Chang HJ, Min S, Woo H, Jennifer Y. Mask-Wearing Behavior During the COVID-19 Pandemic: A Cross-Cultural Comparison Between the United States and South Korea. Familly & Counsumer Sciences. 2021 Aug 22;50(1):5-26.[Doi: 10.1111/fcsr.12416](https://doi.org/10.1111/fcsr.12416)
28. Xu P, Cheng J. Individual differences in social distancing and mask-wearing in the pandemic of COVID-19: The role of need for cognition, self-control and risk attitude. Personality and Individual Differences. 2021 June;175:110706. Doi: 10.1016/j.paid.2021.110706.
29. Hwang J, Chun HR, Cheon E. A qualitative study on the impact of COVID-19 on the behavior and attitudes of smokers and non-smokers in South Korea. BMC Public Health. 2021 Nov 1;21(1):1972. Doi: 10.1186/s12889-021-12079-8.
30. Pokhrel P, Herzog TA, Muranaka N, Fagan P. Young adult e-cigarette users’ reasons for liking and not liking e-cigarettes: A qualitative study. Psychology & Health. 2015 Jul 15;30(12):1450-1469.Doi: [10.1080/08870446.2015.1061129](https://doi.org/10.1080/08870446.2015.1061129)
31. Choi EJ, Lee NH, Yoon SM. Policy Study on Tobacco Regulation and Systematic Management “Korean, author’s translation”. Ministry of Health and Welfare, Korea Institute for Health and Social Affairs. 2018. [cited 2022 May 26]. Available from: <https://www.prism.go.kr/homepage/entire/researchDetail.do?researchId=1351000-201800244>
32. Sawsan A, Karem HA, Omar K. Fear of COVID-19 and stigmatization towards infected people among Jordanian people. The International Journal Of Clinical Practice. 2020 Dec 06;75(4). Doi: [10.1111/ijcp.13899](https://doi.org/10.1111/ijcp.13899)
33. Jackson SE, Brown J, Shahab L, Steptoe A, Fancourt D. COVID-19, smoking and inequalities: a study of 53 002 adults in the UK. BMJ.2020 August 21;30(2). [Doi:/10.1136/tobaccocontrol-2020-055933](http://dx.doi.org/10.1136/tobaccocontrol-2020-055933).
34. Kim K, Jeung YD, Choi J, Park SK. Social and policy determinants of COVID-19 infection across 23 countries: an ecological study. Journal of Preventive Medicine and Public Health. 2022 Feb;*55*(2): 144-152. Doi: [10.3961/jpmph.21.396](https://doi.org/10.3961%2Fjpmph.21.396)
35. Kale D, Herbec A, Perski O, Jackson SE, Brown J, Shahab L. Associations between vaping and Covid-19: cross-sectional findings from the HEBECO study. Drug and Alcohol Dependence. 2021 December 3; 221. [Doi:/10.1101/2020.12.01.20241760](https://doi.org/10.1101/2020.12.01.20241760)
36. Gülsen A, Yigitbas BA, Uslu B,  Drömann D, Kilinc O. The effect of smoking on COVID-19 symptom severity: systematic review and meta-analysis. Hindawi. 2020 sep;2020. Doi: [10.1155/2020/7590207](https://doi.org/10.1155/2020/7590207)
