# Supplementary material for: The association between tobacco or nicotine product use behaviors and non-compliance with mask-wearing during the COVID-19 pandemic: a cross-sectional study in Korea
Source: Epidemiol Health. 2022 Oct 7;44:e2022087. doi: 10.4178/epih.e2022087 (PMC10089704; doi:10.4178/epih.e2022087)
Supplement: Supplementary Material 1. — Adjusted odds ratio(aOR) for not wearing a mask of all participants (N=208,618) [file epih-44-e2022087-Supplementary-1.docx]

**Supplementary Material 1.** Adjusted odds ratio(aOR) for not wearing a mask of all participants (N=208,618)

| Variables | Subgroup | aOR(95% CI)^1^ | |
| --- | --- | --- | --- |
|  |  | Male | Female |
| Tobacco/Nicotine product use status | Current users | 2.00 (1.66, 2.40)* | 1.32 (0.88, 1.99) |
|  | Former users | 1.32 (1.09, 1.60)* | 1.50 (1.00, 2.26)* |
|  | Never users | 1.00(reference) | |
| Age(yr) |  | 1.01 (1.00, 1.01)* | 1.01 (1.01, 1.02)* |
| Occupation | Managers · Professionals | 1.33 (1.01, 1.74)* | 0.88 (0.55, 1.41) |
|  | Clerks | 1.17 (0.87, 1.57) | 1.11 (0.74, 1.65) |
|  | Service and sales workers | 1.35 (1.03, 1.78)* | 1.12 (0.86, 1.46) |
|  | Skilled agricultural and fishery workers | 3.01 (2.47, 3.67)* | 1.92 (1.59, 2.32)* |
|  | Craft · elementary workers | 1.36 (1.12, 1.65)* | 0.90 (0.71, 1.16) |
|  | Unemployed | 1.00 (reference) | |
| Number of household members | Single person | 1.23 (0.98, 1.53) | 1.37 (1.10, 1.71)* |
|  | Multi-person | 1.00 (reference) | |
| Education level | <middle school | 1.65 (1.31, 2.07)* | 1.11 (0.83, 1.48) |
|  | High school | 1.19 (0.99, 1.42) | 0.77 (0.58, 1.02) |
|  | >college | 1.00 (reference) | |
| Region of residence | Rural | 1.55 (1.36, 1.77)* | 2.38 (2.03, 2.80)* |
|  | Urban | 1.00 (reference) | |
| Marital status | Single | 1.09 (0.89, 1.32) | 1.28 (1.05, 1.56)* |
|  | Married | 1.00 (reference) | |
| Monthly household income(Korea won) | <2 million | 0.97 (0.80, 1.19) | 1.01 (0.79, 1.30) |
|  | 2-4 million | 0.97 (0.82, 1.14) | 0.81 (0.65, 1.01) |
|  | >4 million | 1.00 (reference) | |
| ^1^ Adjusted for age, occupation, number of household members, education level, region of residence, marital status, and monthly household income.  * p<0.05. | | | |
