# Supplementary material for: The association between tobacco or nicotine product use behaviors and non-compliance with mask-wearing during the COVID-19 pandemic: a cross-sectional study in Korea
Source: Epidemiol Health. 2022 Oct 7;44:e2022087. doi: 10.4178/epih.e2022087 (PMC10089704; doi:10.4178/epih.e2022087)
Supplement: Supplementary Material 2 — Adjusted odds ratio(aOR) for not wearing a mask of current users (N=36,233) [file epih-44-e2022087-Supplementary-2.docx]

**Supplementary Material 2.** Adjusted odds ratio(aOR) for not wearing a mask of current users (N=36,233)

| Variables | Subgroup | aOR(95% CI)^1^ | |
| --- | --- | --- | --- |
|  |  | Men | Women |
| Tobacco/Nicotine product types | Cigarettes | 1.53 (1.12, 2.08)* | 0.88 (0.29, 2.61) |
|  | NCTNPs | 1.00 (reference) | |
| Changes in TNP use after the COVID-19 outbreak | Increased | 1.22 (0.91, 1.62) | 0.13 (0.07, 0.26)* |
|  | Decreased | 0.52 (0.36, 0.74)* | 0.97 (0.41, 2.29) |
|  | Similar | 1.00 (reference) | |
| Attempt to quit | No | 1.19 (0.98, 1.45) | 0.60 (0.29, 1.25) |
|  | Yes | 1.00 (reference) | |
| Age(yr) |  | 1.02 (1.01, 1.03)* | 1.02 (0.99, 1.05) |
| Occupation | Managers · Professionals | 1.60 (1.10, 2.33)* | 0.84 (0.12, 5.86) |
|  | Clerks | 1.05 (0.67, 1.64) | 1.81 (0.49, 6.62) |
|  | Service and sales workers | 1.20 (0.81, 1.77) | 1.30 (0.50, 3.37) |
|  | Skilled agricultural and fishery workers | 2.94 (2.15, 4.04)* | 3.59 (0.71, 18.25) |
|  | Craft · elementary workers | 1.28 (0.96, 1.71) | 1.00 (0.28, 3.65) |
|  | Unemployed | 1.00 (reference) | |
| Number of household members | Single person | 1.03 (0.78, 1.37) | 3.19 (1.26, 8.11)* |
|  | Multi- person | 1.00 (reference) | |
| Education level | <middle school | 1.65 (1.17, 2.31)* | 0.67 (0.24, 1.89) |
|  | High school | 1.19 (0.93, 1.52) | 1.14 (0.43, 3.02) |
|  | >college | 1.00 (reference) | |
| Region of residence | Rural | 1.34 (1.11, 1.60)* | 1.64 (0.68, 4.00) |
|  | Urban | 1.00 (reference) | |
| Marital status | Single | 1.20 (0.93, 1.54) | 2.33 (0.78, 6.95) |
|  | Married | 1.00 (reference) | |
| Monthly household income(Korea won) | <2 million | 1.13 (0.83, 1.54) | 0.67 (0.25, 1.84) |
|  | 2-4 million | 1.04 (0.82, 1.32) | 0.91 (0.34, 2.42) |
|  | >4 million | 1.00 (reference) | |
| ^1^ Adjusted for age, occupation, number of household members, education level, region of residence, marital status, and monthly household income.  * p<0.05. | | | |
